# Retraction: Changes in Iliopsoas Muscle Activity and Hip/Pelvic Kinematics With Variations in Step Length and Cadence at a Fixed Walking Speed

**DOI:** 10.7759/cureus.r239

**Published:** 2026-08-26

**Authors:** Takumi Jiroumaru, Michio Wachi, Yutaro Hyodo, Yasumasa Oka, Takamitsu Fujikawa

**Affiliations:** 1 Department of Physical Therapy, Bukkyo University, Kyoto, JPN; 2 Department of Rehabilitation, Kanazawa Orthopaedic and Sports Medicine Clinic, Shiga, JPN

This article has been retracted by the Editor-in-Chief. After a review by the author's institution, it was determined that the article substantially overlaps with a previously published article by the same author(s): Jiroumaru T, Hyodo Y, Wachi M, et al. (May 14, 2025) Hip Flexor Muscle Activation Across Gait Phases in Healthy Young Adults: Effects of Step Length and Cadence Adjustments at a Constant Walking Speed. Cureus 17(5): e84130. doi:10.7759/cureus.84130. This constitutes redundant (duplicate) publication, commonly referred to as "salami slicing," which is inconsistent with the journal's authorship and publication ethics policies and with COPE guidelines on redundant publication. The authors agree with the decision to retract.

